# Revascularization Strategy with prolonged cardiopulmonary bypass in a patient with multiple morbidity factors and serious left main coronary artery lesion

**DOI:** 10.1186/1749-8090-10-S1-A222

**Published:** 2015-12-16

**Authors:** Ufuk Yetkin, Nihan Karakaş, Köksal Dönmez, Nagihan Karahan, Ali Gürbüz

**Affiliations:** 1 Department of Cardiovascular Surgery, Katip Celebi University Izmir Ataturk Training and Research Hospital, Izmir, Turkey

## Background/Introduction

Mortality and surgical risk significantly increase in patients who have multiple morbidities and are candidates for surgical coronary revascularization.

## Aims/Objectives

Our case was 71 year-old male patient. He had PTCA ten years ago in his medical history. He was having chest pain episodes for 1 year. In addition, he had stroke 8 years ago and carotid endarterectomy 7 years ago in his medical history. Our patient had morbidity factors for cardiovascular disease, COPD, and benign prostate hypertrophy.

## Method

Coronary angiography and selective arch aortography, revealed plaque formation that was not creating any hemodynamic obstruction on left carotid system and total occlusion of right internal carotid artery. Serious stenosis of 50% at left main coronary artery and three-vessel disease was evident. Interventricular septum was measured as 16 mm in transthoracic echocardiography. High-risk surgical revascularization of coronary arteries was planned.

## Results

After median sternotomy, routine unicaval two stage venous cannulation and aortic cannulation was performed for cardiopulmonary bypass. Mean arterial pressure was stabilized optimally during operation due to risky situation carotid arteries. After ante- and retrograde cardioplegia protocol, cross-clamp was placed. Sequential bypass was performed to PL and PD branches of right coronary artery by using native saphenous vein graft. CxOM1 and LAD were grafted afterwards. Nodal tachycardia occurred while weaning from cardiopulmonary bypass. Due to full inotropic support need, bypass was carried on for myocardial exercise. After re-cross-clamping, fifth distal anastomosis was performed to CxOM2. Normal sinus rhythm was achieved. Patient was transferred to intensive care unit with open sternum and inotropic support (dopamine+dobutamine+norepinephrine) for preventing mediastinal compression and resolution of myocardial edema. Inotropic support need of Patient reduced and he became very awake. He underwent mediastinal re-exploration. All grafts were patent and had optimal configuration. His chest was closed regularly. Patient recovered uneventfully.

## Discussion/Conclusion

Evaluation of high-risk morbidity factors prior to surgical intervention and planning of encountering possible problems will increase the survival rates and safety of procedure.

## Consent

Written informed consent was obtained from the patient for publication of this Case report and any accompanying images. A copy of the written consent is available for review by the Editor-in-Chief of this journal.

